# A Malicious Domain Detection Model Based on Improved Deep Learning

**DOI:** 10.1155/2022/9241670

**Published:** 2022-06-25

**Authors:** XiangDong Huang, Hao Li, Jiajia Liu, FengChun Liu, Jian Wang, BaoShan Xie, BaoPing Chen, Qi Zhang, Tao Xue

**Affiliations:** ^1^Hebei Engineering Research Center for the Intelligentization of Iron Ore Optimization and Ironmaking Raw Materials Preparation Processes, North China University of Science and Technology, Tangshan, Hebei, China; ^2^Hebei Key Laboratory of Data Science and Application, North China University of Science and Technology, Tangshan, Hebei, China; ^3^The Key Laboratory of Engineering Computing in Tangshan City, North China University of Science and Technology, Tangshan, Hebei, China; ^4^College of Science, North China University of Science and Technology, Tangshan, Hebei, China; ^5^Tangshan Intelligent Industry and Image Processing Technology Innovation Center, North China University of Science and Technology, Tangshan, Hebei, China; ^6^College of Qian An, North China University of Science and Technology, Tangshan, Hebei, China

## Abstract

With the rapid development of the Internet, malicious domain names pose more and more serious threats to many fields, such as network security and social security, and there have been many research results on malicious domain detection. This article proposes a malicious domain name detection model based on improved deep learning, which can combine the advantages of three different network models, convolutional neural network (CNN), temporal convolutional network (TCN), and long short-term memory network (LSTM) in malicious domain name detection, to obtain a better detection effect than that of the original single or two models. Experiments show that the effect of the improved deep learning model proposed in this article is better than that of the combined model of CNN and LSTM or the combined model of CNN and TCN, and the accuracy and regression rates reached 99.76% and 98.81%, respectively.

## 1. Introduction

In the information age with the continuous updating and iteration of Internet technology, the rapidly developing network brings convenience to the people, and the threat means hidden in the network emerge one after another. Phishing websites, botnets, and other network attacks that rely on domain name jump technology, fast-flux [1], and other technical means to avoid security detection pose a serious threat to network information security, personal privacy security, social property security, and other aspects. This is precisely because the threat of malicious domain names to network security is becoming more and more serious, which also makes the research on the detection of malicious domain names tend to be hot. Researchers have also detected malicious domain names from many different angles.

In the detection methods of malicious domain names, analyzing and extracting the character-level features in the string of domain names to identify the legitimacy of domain names is one of the common methods. It mainly analyzes the difference between the distribution law of the string used by the legal domain name and the normal domain name and uses a certain method to obtain the character-level characteristic law implied in the string to judge whether it is a malicious domain name, For example, the domain name string is analyzed from the aspects of the proportion of meaningful characters [2], morpheme features, etc. in the current research results, there are mainly two categories of character-level feature extraction for domain names. One is to extract the features of the string data of the domain name dataset by manually writing functions. The effect of feature extraction depends on the feature extraction function used. The other is to use relevant algorithms such as neural networks in machine learning or their improved algorithms to realize the automatic extraction of character-level features of domain name data and subsequent analysis, processing, and discrimination [3–5]. The detection effect of such methods is often affected by the model structure of the neural network, and the detection accuracy of the model depends on a large number of training datasets. In addition, some researchers identify the characteristics of malicious domain names by analyzing DNS traffic data. Researchers use many detection methods; for example, K-means algorithm and smote method are combined [6], convolutional neural network structure and cyclic neural network are combined to detect malicious domain names involved in botnets [7], and RBF kernel is added to support vector machine algorithm to improve the detection effect of malicious domain names [8].

Due to the difference in string length distribution between normal domain names and malicious domain names, some researchers established a malicious domain name detection model [9], analyzed and compared the distribution of character types used in the domain name [2], and used Jaccard coefficient [10], K-L distance, and other different statistical features to identify the legitimacy of the domain name. Some researchers also use graph neural network to determine the nature of domain names by modeling DNS scenarios [11]. In order to improve the model's real-time online detection of malicious domain names, Yang Lu Hui et al. proposed a fast3DS real-time malicious domain detection system that includes a lightweight full convolutional network [12]. In order to solve the shortcomings of static blacklists, researchers used a combination of LSTM and adversarial machine learning algorithms to build malicious domain name classifiers [13]. Some researchers also established a system called Mal-Portrait and used the individual characteristics and associated characteristics of the domain name and detected malicious domain names [14]. Through the analysis and comparison of DNS data obtained by active domain names or passive domain names [15] and the use of machine learning algorithms, the detection of malicious domain names can also be achieved. In the study of DNS analysis, some researchers have developed an algorithm called IMDOC, which can analyze the association between malicious domain names and actual DNS traffic [16]. In improving the model's ability to detect fast-flux domain names, some researchers have improved the detection ability using traffic anomaly filtering and association matching algorithms [17]. Some researchers also use a pretrained word vector model and the Text-CNN model to detect malicious domain names [18].

To sum up, among the existing malicious domain name detection models, many improvement methods for CNN detection models mostly improve the detection effect by improving the model's character-level feature extraction without further mining malicious domain name data and time. Many DGA algorithms usually use network time as a seed when generating domain name string data. Therefore, the detection effect of the model can be further improved by mining the relationship between malicious domain name data and time series. This article mainly considers two aspects to optimize the detection effect of the model: one is to add an LSTM layer network structure with an attention mechanism to the original CNN model [19] so as to increase the original model's ability to detect domain name strings. While detecting the distribution features of possible word combinations, improve the detection dimension of the model for character-level features; the second is to add a TCN network structure to the CNN convolutional layer and improve the original CNN network with the help of the characteristics of the TCN network structure. Experiments show that by adding TCN network structure and LSTM network with an attention mechanism to the original CNN network structure, the detection accuracy of the model for malicious domain names can be improved to a certain extent. In the comparative experiments of various methods, the comprehensive performance of the malicious domain name detection model proposed in this article is the best, with an accuracy rate of 99.12%, a regression rate of 99.76%, an accuracy rate of 98.81%, and a false-positive rate of 1.52%.

## 2. Model Structure

### 2.1. The Basic Structure of the Detection Model

**Figure 1:**
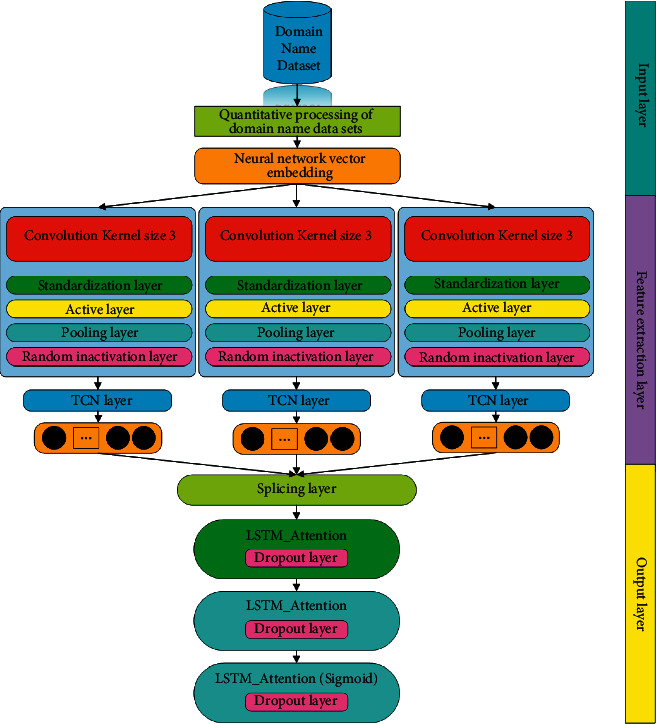
Basic structure of detection model.

The malicious domain name detection model constructed in this article optimizes the model and improves the detection accuracy by adding TCN network structure and LSTM network with attention mechanism on the basis of CNN network structure. The malicious domain name detection model constructed in this article mainly identifies the nature of malicious domain names by extracting the character-level features of domain name datasets. Its basic structure is shown in Figure 1.

**Figure 2:**
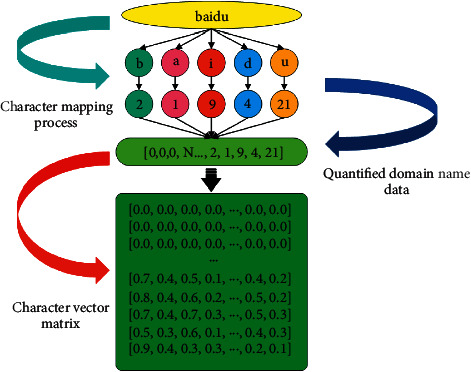
Basic principles of character encoding.

Similar to most text data processing models, the malicious domain name detection model constructed in this article also includes three parts: input layer, feature extraction layer, and result output layer. The basic function of the input layer is to encode and convert the domain name dataset. In terms of domain name character quantization, it is more common to use single hot coding [20] and distributed representation model, and a few researchers use ASCII coding. In comprehensive consideration and comparison of the advantages and disadvantages of the three-domain name character quantization, the main character encoding method used in the detection model of this article is the distributed representation model, and its basic principle is shown in Figure 2. It can better avoid the problems that the data dimension is too high and it is difficult to express the possible internal relations in different length strings in the unique coding method and ASCII coding method and has better comprehensive performance.

It converts the domain name data into a data matrix according to the basic characteristics of characters and normalizes the converted matrix data. The feature extraction layer is the basic body of the whole malicious domain name detection model; it is divided into three convolution layers according to different convolution core sizes, namely, the convolution network with convolution core sizes of 3, 4, and 5. Each convolution network has two identical convolution layers, and each convolution layer includes a convolution layer, standardization layer, activation layer, and pooling layer. In order to improve the effectiveness of the model in the extraction of association features between domain name characters and improve the flexibility of the network receptive field, a time convolution network (TCN) is added behind each convolution network. Then input the feature extraction results of each convolutional network into the unfolding layer, perform network splicing, and then enter the result output layer containing the fully connected layer. The result output layer mainly contains a layer of LSTM with attention mechanism and two layers of fully connected layers with different numbers of neurons.

### 2.2. CNN Network Structure

A convolutional neural network (CNN) has long been used by researchers in text classification tasks and achieved good classification results. Many researchers have applied the CNN network and its improvements to text classification tasks [21–24] and achieved good results. Since malicious domain name detection is essentially a text classification task, it can also be used to detect malicious domain names. Studies have shown that the feature extraction efficiency of convolution kernel size *n* used in the convolution neural network is similar to that of the N-gram model structure [25] in natural language processing, but the overall feature extraction efficiency is higher. In order to increase the feature extraction effect of different length string combinations in the domain name string, in the malicious domain name detection model structure used in this article, the convolution core sizes of 3, 4, and 5 are used to extract the 3-gram, 4-gram, and 5-gram features contained in the domain name string, respectively.

### 2.3. LSTM Layer Structure with Attention Mechanism

LSTM can mine the internal relationship between different characters in string data and can achieve different practical results in tasks, such as sequence analysis, natural language processing, and speech recognition. In the process of sequence analysis and natural language processing, due to its long context, the LSTM network model can optimize the originally used N-gram model and standard recursive network model [26]. After adding the attention mechanism, the number of parameters to be trained can be further reduced, and the distribution of sequence parameter weights during training can be enhanced [27]. In the task of speech recognition, the use of a bidirectional short-term memory network (BLSTM) can make full use of the context information in speech data, and the PSRBL network added to the BLSTM network can reduce the time of model training and recognition [28].

**Figure 3:**
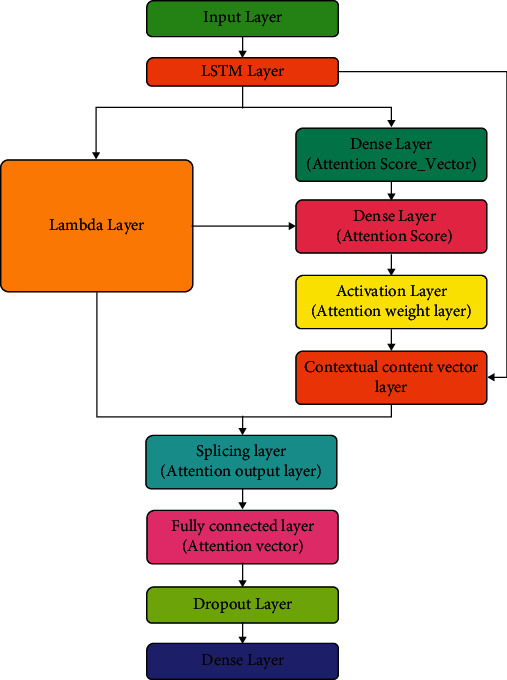
LSTM combined with an attention mechanism.

The original LSTM network structure shows some problems in text classification, such as imperfect vector representation structure of model learning output and a large amount of calculation when facing a large time span. Adding attention mechanism to the LSTM network structure can optimize the vector representation results obtained by the LSTM structure to a certain extent, improve the overall efficiency of the network model structure and reduce the training time while making full use of the correlation between the front and back text in the string. The basic principle of the attention mechanism used in this article is shown in Figure 3.

As shown in the figure, after the dataset is input and passes through the LSTM layer network, it enters the hidden layer structure representing the state defined by lambda and the network structure used to process and calculate the attention weight. In the obtained attention weight structure, after passing through two fully connected layer networks, the attention score vector and the final attention score are obtained, respectively, and the attention weight is obtained. Its calculation formula is shown in the following formula:

(1) $$\begin{array}{c} \alpha_{ts}=\frac{\exp\left(\text{score}\left(h_{t},h_{s}\right)\right)}{\sum_{S^{\prime }=1}^{S}\exp\left(\text{score}\left(h_{t},h_{s^{\prime }}\right)\right)}. \end{array}$$

Among them, *α*_*ts*_ indicates the attention weight parameter value; *h*_*t*_ and *h*_*s*_ represent the hidden measurement output at time *t* and the hidden layer output of the source statement, respectively, and S represents the semantics of the source statement. On the basis of obtaining the attention weight parameter value, continue to enter the next layer network and obtain the context content vector *C*_*t*_. Its calculation process is shown in the following formula:

(2) $$\begin{array}{c} C_{t}=\sum_{S}\alpha_{ts}h_{s}. \end{array}$$

After the splicing layer, the attention vector is obtained after entering the full connection layer. The calculation method of the attention vector is shown in the following formula:

(3) $$\begin{array}{c} a_{t}=f\left(c_{t},h_{t}\right)=\tanh\left(W_{c}\left[C_{t}:h_{t}\right]\right). \end{array}$$

Among them, *a*_*t*_ represents the attention vector parameter, *C*_*t*_ represents the context content vector, and *h*_*t*_ represents the hidden layer output of time *t*. After obtaining the attention vector, it enters the random deactivation layer and the full connection layer network structure to obtain the output data of the LSTM layer network structure with the attention mechanism.

### 2.4. TCN Layer Network Structure

The time convolution network (TCN) can deal with time series better than the ordinary convolution network and can mine the implied association between datasets and time series. Researchers have applied TCN networks in many different fields, such as weather forecast calculation [29] and greenhouse crop yield prediction [30].

In most malicious domain name detection models, the relationship between domain name dataset and time series is often ignored. Through data statistical analysis, it is shown that there is a certain correlation between the time distribution before and after the emergence of new malicious domain names, and the time distribution of different kinds of DGA domain names is also different. Therefore, adding the TCN network structure to the original detection model can help the model mine the relationship between the malicious domain name itself and the time series, thereby improving the detection effect of the model.

**Figure 4:**
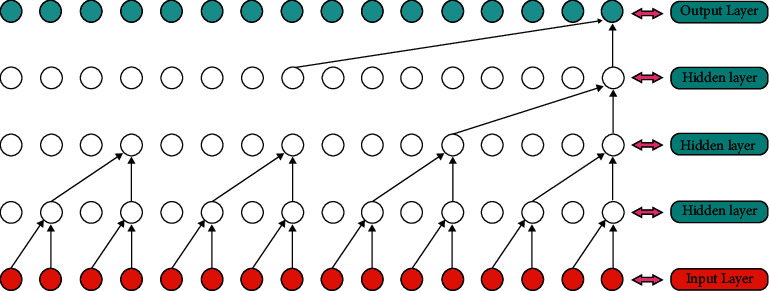
Basic structure diagram of TCN (expanded convolution).

Temporal convolutional networks (TCN) are roughly divided into three types, namely, causal convolution [31], dilated convolution, and residual connections. They are in the network structure and there are certain differences in classification effects. The TCN network structure used in the detection model in this article is mainly dilated convolution, and its basic structure is shown in Figure 4.

Since the expanded convolution structure uses interval sampling for the output parameters, the sampling size is controlled by the sampling distance *d*. The model in the figure uses *d* = 2; that is, the output sampling of every two points is used as an input, and the higher the level *d* will also be larger, which also enables this network to obtain a larger receptive field while using fewer layers, increasing the data sampling rate of the model.

## 3. Experiment and Result Analysis

### 3.1. Research Methods and Experimental Design

In order to test the practical effect of the malicious domain name detection model proposed in this article, we will use public datasets from the network as the experimental data used in the experiments. They come from a part of the dataset on Alex's website and a dataset composed of 54 DGA domain name data published by 360 Network Lab, and they are divided into the training set, validation set, and test set, in which the ratio of the training set and test set is 10 : 1 and contains 153,714 data, while the test set of the experiment is a total of 20,000 data randomly selected from the remaining domain datasets. During the experiment, the detection model is trained using the prelabeled training set and validation set data so that the various parameters of the model are continuously adjusted to the best state. Finally, the training effect of the model is tested by the unlabeled test set data.

**Figure 5:**
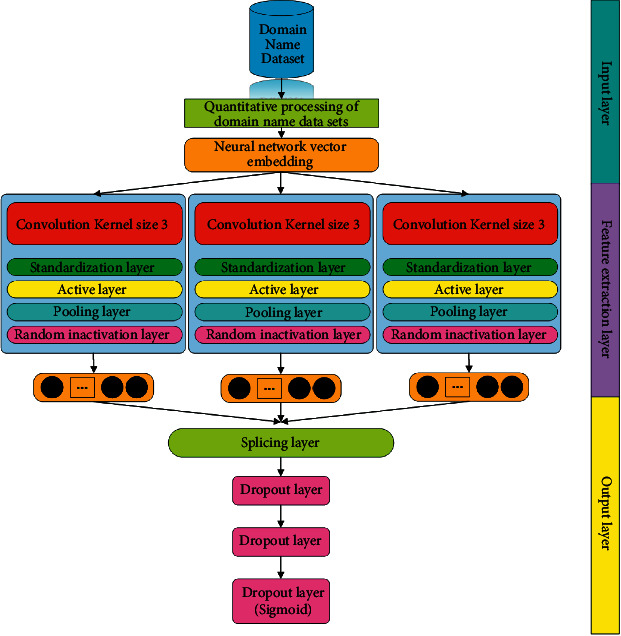
Basic CNN network model.

**Figure 6:**
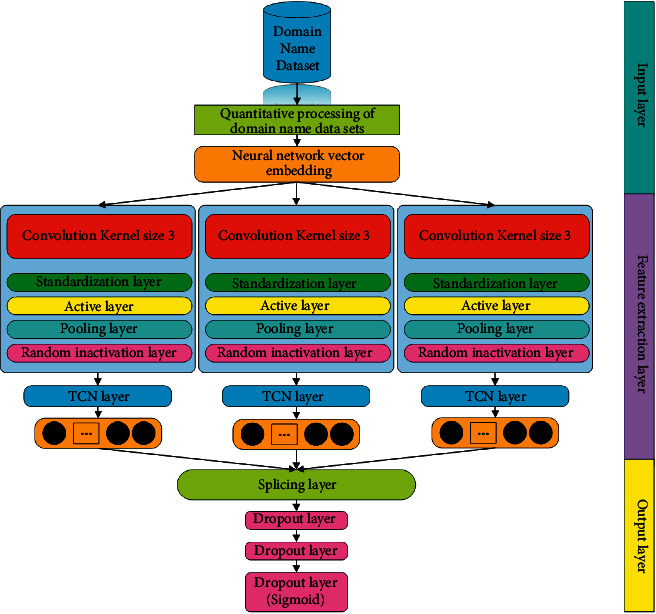
CNN + TCN network model.

**Figure 7:**
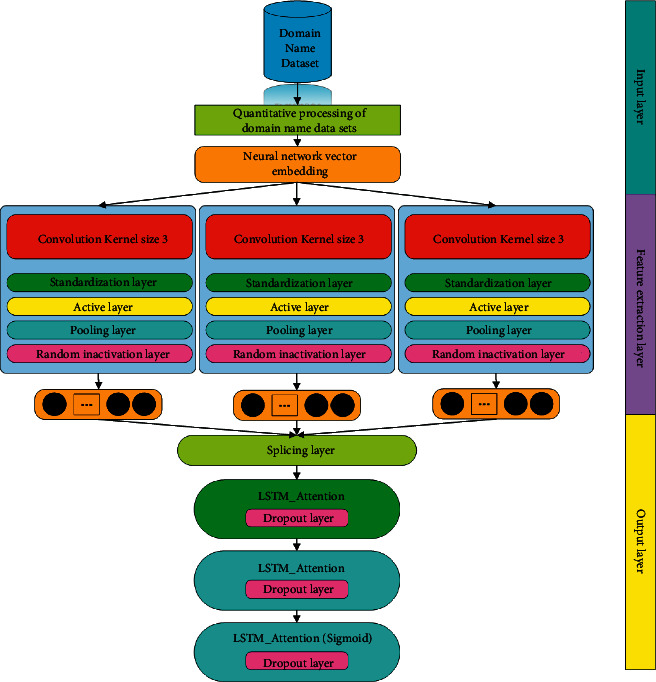
CNN + LSTM with an attention mechanism.

In order to observe the actual effect of the model proposed in this article, this article also simultaneously designs the more common models in malicious domain name detection and their improved models and uses their experimental results as comparative experiments. The comparative experimental models are the CNN model, a combined model of the network and an LSTM network with an attention mechanism, and a combined model of a CNN network and a TCN network. The basic structure of the comparative experimental model used in this article will be described in Figures 5, 6, and 7 in detail.

As can be seen from the contents of Figures 5, 6, and 7, there are only a few differences between the basic structures of the three comparative experimental models and the basic structure of the model proposed in this article. This design is also for comparing their experimental results, which become more meaningful. In the evaluation of the experimental results, the evaluation indicators that are commonly used in the current model evaluation of malicious domain name detection are used in this article. The evaluation parameters of the experiment include various parameters in the confusion matrix, correct rate, precision rate, and recall rate. We will conduct a specific analysis through the results of these parameter indicators of each model. In addition, this article will compare the time complexity and complexity of different models and further compare the performance parameters of different models in depth.

### 3.2. Dataset Selection and Processing

The experimental data sources used in the experimental model in this article mainly have two parts. One part comes from the domain name data of the top one million in the Alexa global domain name ranking, and 30,000 domain names are taken as the normal domain name dataset for training data. The other part comes from the DGA domain name data published by 360 Network Lab. A total of 54 DGA domain name datasets are collected through the collection. Their types and data distribution are shown in Table 1.

Because the number of some types of domain name datasets is too small, which is not conducive to the experiment, we select the DGA domain names whose malicious domain data volume is greater than 10,000, obtain a total of 11 DGA domain name datasets, as shown in Table 2, and use them as training for the malicious domain name dataset in the data. The experimental dataset is sorted to the end, a total of 110,000 normal domain name datasets and 110,000 malicious domain name datasets are input into the model, and training is carried out at a ratio of 10 : 1 between the training set and the verification set. Moreover, after saving the trained model, some data samples will be selected from the remaining normal domain name dataset and malicious domain name dataset as the test set of the model.

Regarding the data preprocessing part of the experimental model, since the collected and obtained data samples contain some irrelevant information from the original website, it is necessary to delete and filter the original dataset to obtain a pure domain name dataset. In addition, in addition to the quantitative processing of the domain name dataset input to the model, in order to facilitate experimental testing and data statistics, the dataset is normalized.

### 3.3. Description of Experimental Environment and Experimental Evaluation Indicators

The basic conditions of the experimental environment used in the malicious domain detection model in this article: the Python version used is 3.8.8, the third-party libraries mainly include Keras and TensorFlow, the operating system of the hardware platform used in the experiment is Window 10, the processor is AMD Ryzen 5 4600H with Radeon Graphics 3.00 GHz, and the machine with RAM is 16.0 GB.

The model evaluation parameters used in this article mainly include precision rate, recall rate, F1 parameter index, false-positive rate, and true-positive rate, which can well reflect the actual classification effect of the model. The ROC curve and PR curve are used to analyze their performance, and the area under the curve (AUC) can evaluate the model's binary classification performance as a whole. Their calculation method is shown in the following formula:

(4) $$\begin{array}{c} A_{\text{avg}}=\frac{A_{\text{pos}}+A_{\text{neg}}}{2},\\ R=\frac{N_{\text{TP}}}{N_{\text{TP}}+N_{\text{FN}}},\\ V_{\text{AUC}}=\frac{\sum I\left(P_{\text{pos}},P_{\text{neg}}\right)}{\text{MN}},\\ P=\frac{N_{\text{TP}}}{N_{\text{TP}}+N_{\text{FP}}},\\ \text{FPR}=\frac{N_{\text{TP}}+N_{\text{TN}}}{N_{\text{TP}}+N_{\text{FP}}+N_{\text{TN}}+N_{\text{FN}}},\\ I\left(P_{\text{pos}},P_{\text{neg}}\right)=\left\{\begin{array}{c} 1,P_{\text{pos}}>P_{\text{neg}},\\ 0.5,P_{\text{pos}}=P_{\text{neg}}\\ 0,P_{\text{pos}}<P_{\text{neg}}, \end{array}\right.,\\ F1-\text{Score}=\frac{2\times \text{Precison}\times \text{Recall}}{\text{Precision}+\text{Recall}}. \end{array}$$

In the above formula, *A*_avg_ is the average accuracy, *A*_neg_ is the average accuracy, *A*_neg_ is the accuracy of negative sample classification, *R* is the recall rate, *N*_TP_ is the number of false-positive samples, *V*_AUC_ is the area under the curve, *P*_pos_ is the probability that the prediction is a positive sample, *P*_neg_ is the probability that the prediction is a negative sample, *M* is the number of positive samples, *N* is the number of negative samples, and FRP is the false alarm rate and it can well reflect the feasibility of the model judgment result. *F*1 − Score means the grade of *F*1; the larger the value, the better the performance of the model.

In addition to the experimental test of the model designed in this article, the experiment also designed a part of the test model for comparative experiments. The experimental comparison models include CNN model, CNN network and LSTM network combined with attention mechanism model, CNN network combined with the TCN network model, and the experimental model built in this paper, and multiple evaluation indicators are used in the analysis of experimental results to evaluate their performance.

### 3.4. Comparison and Analysis of Results

In order to verify the actual performance of the detection model set in this article on the malicious domain name dataset, a total of 220,000 domain name datasets set in advance are used as the model input, and the dataset is preprocessed before the dataset is input to the model, including the use of a distributed model to quantify the domain name dataset and to scramble the dataset to avoid the uneven distribution of the dataset from affecting the training effect of the model. The same processed dataset is used as the input of different detection models, and their training effects are compared after the same processing. The relevant results of the first test are shown in Table 3.

In the above table, Precision refers to the accuracy of the model, Recall refers to the regression rate of the model, Accuracy refers to the accuracy of the model, FPR refers to the false alarm rate of the model, and F1-Score refers to the model's F1 score.

From the data in the table, it can be seen that in the detection of malicious domain names, the pure CNN network model is slightly inferior to other models in terms of the performance of the detection effect, and the TCN network structure is added to the original CNN network model or added with attention. The LSTM network structure of the force mechanism can improve the detection effect of the original model to a certain extent. Adding LSTM with an attention mechanism to the original CNN network structure can enhance the model's edge detection capabilities for text sequences in domain name strings, while adding TCN network structure can mine the domain name string data and time series. The implicit relationship between the two can improve the detection effect of the model. From the comparison of the performance indicators of the different models in the table, it can be seen that after combining the TCN network structure and the LSTM network structure with the attention mechanism, the composite network model has a detection effect on the malicious domain name dataset; the best detection accuracy and precision reached 99.12% and 98.35%, respectively. It also shows that both the TCN network structure and the LSTM network with attention mechanism can improve the detection performance of the model to varying degrees.

**Figure 8:**
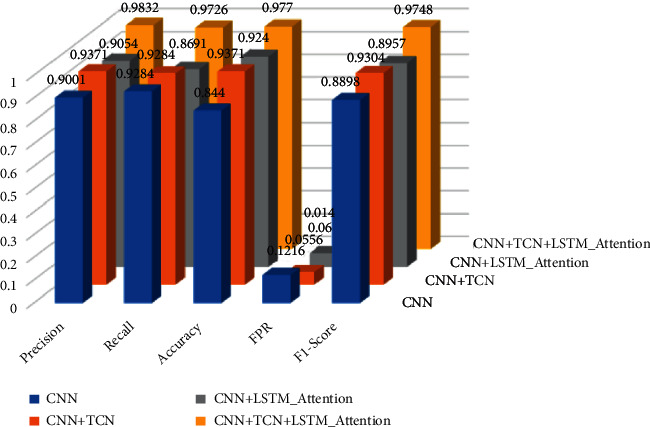
Average values of different parameter indexes of different models (repeated five times).

In order to more objectively reflect the actual performance of different models in the predetermined test set, all models are tested on the test set after the same number of iterations, and this process is repeated 5 times, and the average of the results is taken. The specific conditions of the different indicators of the model are shown in Figure 8.

**Figure 9:**
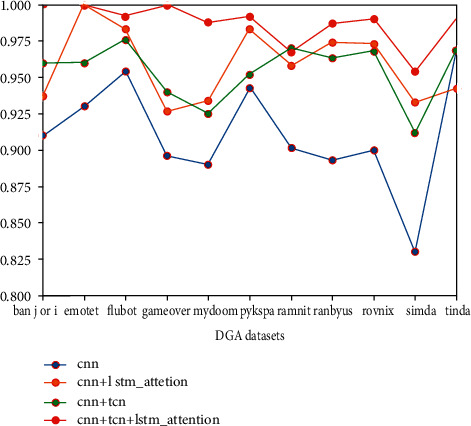
Model checking effect comparison chart.

In addition to evaluating the effect of the model from the comparison of various indicators, the experiment also compared their actual effects by recording the detection accuracy of different models on different types of DGA datasets. In order to avoid being affected by the test dataset and the training data duplication, the dataset used in this round of experiments is the domain name data after removing the data used in the training set. A total of 153714 datasets of 11 domain names are included. The test set is composed of the DGA domain name dataset. As shown in Figure 9, it records in detail the detection effects of different models in all 11 types of DGA datasets.

The data in the figure records the detection accuracy of different detection models in different DGA datasets. The relevant data in the table can explain that the TCN network structure or the LSTM network structure with attention mechanism is added on the basis of CNN. It can improve the detection effect of the original CNN network on the malicious domain name dataset to a certain extent, and the detection model adopted in this article uses two different network structures to combine with the CNN network. The detection effect is the best, and it also proves that the model built in this article is effective.

**Figure 10:**
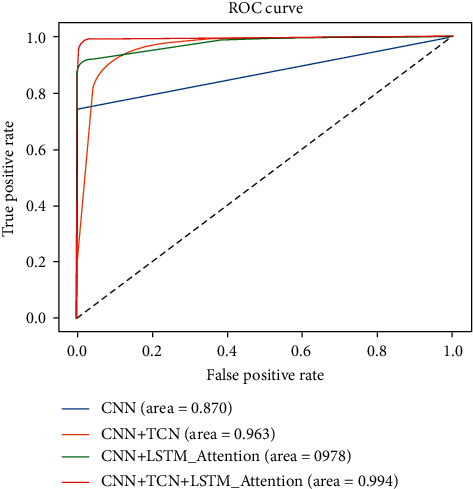
ROC curve of model.

**Figure 11:**
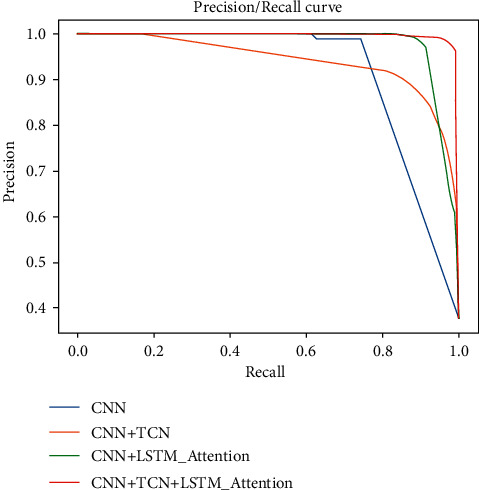
PR curve of model.

Because the ROC curve and AUC parameters can well explain the overall performance superiority of a binary classification model, AUC refers to the area surrounded by the ROC curve and coordinate axis. The larger its value, the better the performance of the model. Similarly, the comparison between PR curves of different models can also reflect the performance differences between different models. The ROC curves and PR curves of the four different models tested in this article are shown in Figures 10 and 11.

From the curve performance in the figure and the comparison of its AUC area, it can be seen that the AUC value of the CNN model is the smallest, which is 0.870, while the AUC value of adding TCN network structure or LSTM network structure with an attention mechanism to CNN structure has increased, and the detection model established in this article has the largest AUC parameter value, which reaches 0.994. It also shows the superiority of the model established in this article.

**Figure 12:**
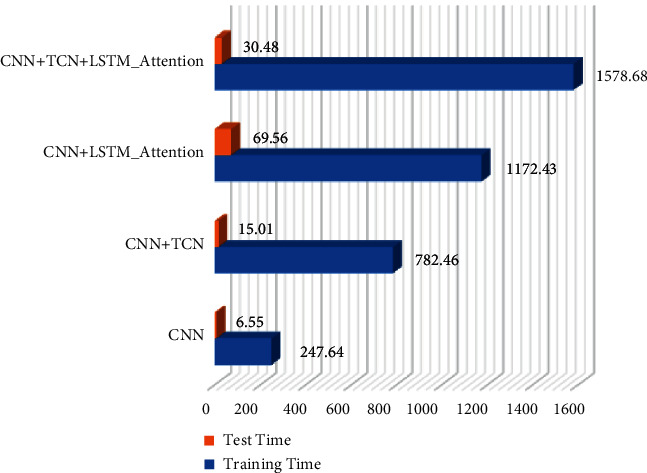
Comparison chart of operation time of different network models.

The running rate of the detection model can also show the excellent performance of the algorithm to a certain extent. After the main experiments in this article are basically completed, an additional experiment on the running time of different detection models is added, although the detection model mentioned in this article is not the best in the running time performance. In the experiment of testing the operation time of the model, a total of 153,714 DGA domain name datasets, including 11 domain name datasets, are used as the training dataset to measure the test time, and a total of 22,000 domain name data, including 11 types of domain names and normal domain names, are used as the measurement training time test dataset. In the experiment, each model iterates the training data for the same round, saves various parameters of the model, and loads and runs the saved model parameters in the calculation of the test set. The specific results are shown in Figure 12.

From the experimental results in Figure 12, it can be seen that the testing time and training time of the pure CNN detection model are the shortest, and the model established in this article has the longest training time. It is caused by the LSTM network structure and TCN network structure of the attention mechanism. The addition of the two network structures can improve the detection effect of the model on malicious domain names, but it also increases the complexity of the model to a certain extent, so it takes the longest time in the training process. Although the operation time of the model is long, the effect of the model established in this article is still the best.

## 4. Summary and Prospect

Due to the security risks caused by malicious domain names to network security, social security, and other aspects, how to improve the detection of malicious domain names has become a problem of great practical significance and application value. Some researchers have shown that the LSTM network structure with attention mechanism can improve the detection effect of the model on malicious domain names, and the relationship between malicious domain name dataset and time series has always been ignored in the detection methods of malicious domain names. This article proposes an improved model based on CNN. Adding TCN network structure and LSTM network structure with attention mechanism can increase the relationship between the model and malicious domain name dataset and time series and improve the detection effect of the model at the character level. The detection model proposed in this article does not need to manually extract features like other detection models based on a deep learning algorithm; it is also improved to a certain extent on the basis of some original detection models. Although the model in this article can improve the detection performance compared with some previous models, there are still some problems in the model, such as long training time and strong dependence on the size of the dataset. The subsequent research and improvement will focus on optimization in this regard.

## Figures and Tables

**Table 1:** 54 DGA domain name datasets.

Label	DGA	Number
1	Abcbot	27
2	Antavmu	16
3	Bamital	104
4	Banjori	483084
5	Bigviktor	1000
6	Blackhole	2
7	Ccleaner	1
8	Chinad	1000
9	Conficker	498
10	Copperstealer	11
11	Cryptolocker	1000
12	Dircrypt	766
13	Dyre	1000
14	Emotet	496892
15	Enviserv	492
16	Feodo	263
17	Flubot	30000
18	Fobber	597
19	Gameover	12000
20	Gspy	100
21	Kfos	124
22	Locky	1158
23	Madmax	1
24	Matsnu	905
25	Mirai	1
26	Monerominer	0
27	Murifet	8560
28	Mydoom	10049
29	Necro	2708
30	Necurs	8188
31	Ngioweb	5275
32	Nymaim	480
33	Omexo	38
34	Padcrypt	168
35	Proslikefan	100
36	Pykspa	45670
37	Qadars	2000
38	Ramnit	20065
39	Ranbyus	11160
40	Rovnix	179993
41	Shifu	2545
42	Shiotob	8004
43	Simda	30289
44	Suppobox	2269
45	Symmi	4256
46	Tempedreve	193
47	Tinba	100653
48	Tinynuke	32
49	Tofsee	20
50	Tordwm	510
51	Vawtrak	842
52	Vidro	100
53	Virut	9748
54	Xshellghost	1

**Table 2:** DGA domain name dataset selection of the experimental model.

Label	DGA	Number	Input dataset size (training set + validation set)	Test set
1	Banjori	483084	10000	20000
2	Emotet	496892	10000	20000
3	Flubot	30000	10000	20000
4	Gameover	12000	10000	2000
5	Mydoom	10049	10000	49
6	Pykspa	45670	10000	20000
7	Ramnit	20065	10000	10065
8	Ranbyus	11160	10000	1160
9	Rovnix	179993	10000	20000
10	Simda	30289	10000	20000
11	Tinba	100653	10000	20000

**Table 3:** Comparison table of various performance indicators of each model.

	Precision (%)	Recall (%)	Accuracy (%)	FPR (%)	F1-score
CNN	88.94	94.99	82.35	15.74	0.8822
CNN + TCN	94.16	94.99	91.50	6.44	0.9321
CNN + LSTM_Attention	92.61	86.92	92.61	3.77	0.8967
CNN + LSTM_Attention + TCN	99.12	99.76	98.81	1.52	0.8935

## Data Availability

The data used to support the findings of this study are available from the corresponding author upon request.
